# C151 in KEAP1 is the main cysteine sensor for the cyanoenone class of NRF2 activators, irrespective of molecular size or shape

**DOI:** 10.1038/s41598-018-26269-9

**Published:** 2018-05-23

**Authors:** Sharadha Dayalan Naidu, Aki Muramatsu, Ryota Saito, Soichiro Asami, Tadashi Honda, Tomonori Hosoya, Ken Itoh, Masayuki Yamamoto, Takafumi Suzuki, Albena T. Dinkova-Kostova

**Affiliations:** 1 Jacqui Wood Cancer Centre, Division of Cancer Research, School of Medicine, University of Dundee, Dundee, Scotland United Kingdom; 2 Department of Medical Biochemistry, Tohoku University Graduate School of Medicine, Aoba-ku, Sendai Japan; 3 Department of Chemistry and Institute of Chemical Biology & Drug Discovery, Stony Brook University, Stony Brook, NY 11794-3400 USA; 4 Department of Stress Response Science, Hirosaki University Graduate School of Medicine, Hirosaki, Japan; 5 Department of Pharmacology and Molecular Sciences and Department of Medicine, Johns Hopkins University School of Medicine, Baltimore, MD USA

**Keywords:** Mechanism of action, Pharmacology

## Abstract

Numerous small molecules (termed inducers), many of which are electrophiles, upregulate cytoprotective responses and inhibit pro-inflammatory pathways by activating nuclear factor-erythroid 2 p45-related factor 2 (NRF2). Key to NRF2 activation is the ability to chemically modifying critical sensor cysteines in the main negative regulator of NRF2, Kelch-like ECH-associated protein 1 (KEAP1), of which C151, C273 and C288 are best characterized. This study aimed to establish the requirement for these cysteine sensor(s) for the biological activities of the most potent NRF2 activators known to date, the cyclic cyanoenones, some of which are in clinical trials. It was found that C151 in KEAP1 is the main cysteine sensor for this class of inducers, irrespective of molecular size or shape. Furthermore, in primary macrophage cells expressing C151S mutant KEAP1, at low concentrations, the tricyclic cyanoenone TBE-31 is inactive as an activator of NRF2 as well as an inhibitor of lipopolysaccharide-stimulated gene expression of the pro-inflammatory cytokines IL6 and IL1β. However, at high inducer concentrations, NRF2 activation proceeds in the absence of C151, albeit at a lower magnitude. Our findings highlight the intrinsic flexibility of KEAP1 and emphasize the critical importance of establishing the precise dose of NRF2 activators for maintaining on-target selectivity.

## Introduction

The KEAP1/NRF2/ARE pathway is at the forefront of cellular defense. Induction of this pathway is protective against various conditions of stress. Conversely, failure to upregulate the pathway leads to increased susceptibility and accelerated disease pathogenesis. Under basal conditions, transcription factor nuclear factor-erythroid 2 p45-related factor 2 (NRF2) is targeted for ubiquitination and proteasomal degradation by a repressor protein Kelch-like ECH-associated protein 1 (KEAP1)^1,2^, a substrate adaptor for Cullin 3 (CUL3)-based E3 ubiquitin ligase^3–5^. Pharmacological inducers of the pathway chemically modify specific cysteine residues within KEAP1, leading to loss of repressor function, NRF2 accumulation and enhanced transcription of genes encoding a large network of cytoprotective proteins^6–10^. The NRF2 target genes contain specific regulatory sequences, termed antioxidant response elements (ARE) in their promoter/enhancer regions, to which NRF2 binds as a heterodimer with a small MAF transcription factor. The products of these genes control various antioxidant pathways such as glutathione (GSH) synthesis and regeneration [e.g. glutamate-cysteine ligase catalytic subunit (GCLC) and glutamate-cysteine ligase modifier subunit (GCLM)], GSH utilization [e.g. glutathione S-transferases (GST)], thioredoxin synthesis and regeneration [e.g. thioredoxin 1 (TXN1), thioredoxin reductase (TXNRD1) and peroxiredoxin 1 (PRDX1)] and NADPH production [e.g. glucose 6-phosphate dehydrogenase (G6PD), 6-phosphogluconate dehydrogenase (PGD), malic enzyme (ME1), isocitrate dehydrogenase (IDH1)]. The enzyme NAD(P)H:quinone oxidoreductase 1 (NQO1), a highly inducible prototypic NRF2 target, is an NAD(P)H-dependent flavoprotein with diverse cytoprotective activities^11^. NQO1 catalyzes the obligatory 2-electron reductions of quinones, thus preventing glutathione depletion and redox cycling.

**Figure 1:**
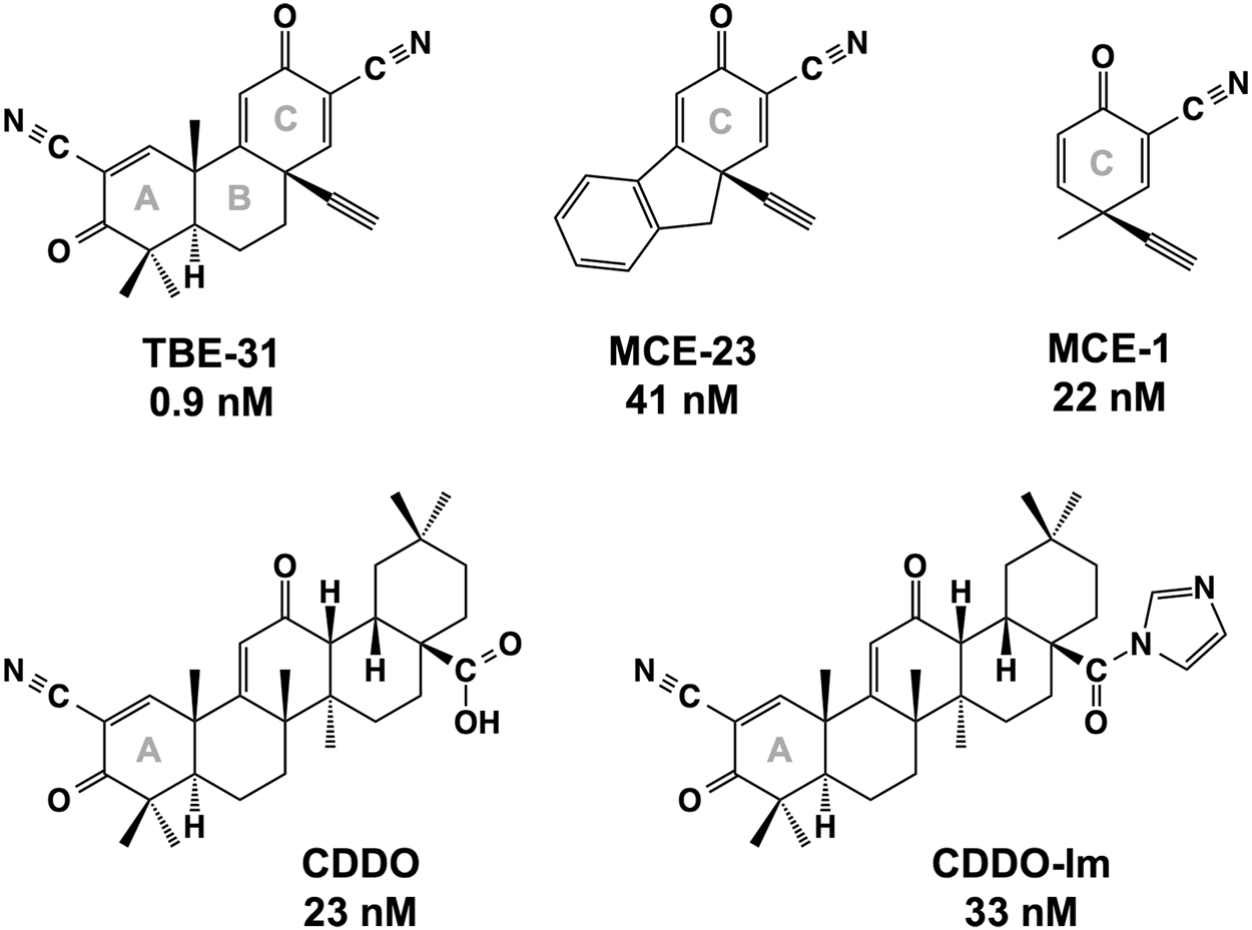
Selected cyanoenone NRF2 activators and their NQO1 inducer potencies (CD values) in Hepa1c1c7 cells. Chemical structures of: 2-cyano-3,12-dioxooleana-1,9(11)-dien-28-oic acid (CDDO), 2-cyano-3,12-dioxooleana-1,9(11)-dien-28-oic acid (CDDO-Im), (±)-(4b*S*,8a*R*,10a*S*)-10a-ethynyl-4b,8,8-trimethyl-3,7-dioxo-3,4b,7,8,8a,9,10,10a-octahydrophenanthrene-2,6-dicarbonitrile (TBE-31), 9a-ethynyl-3-oxo-9,9a-dihydro-3*H*-fluorene-2-carbonitrile (MCE-23), and 3-ethynyl-3-methyl-6-oxocyclohexa-1,4-dienecarbonitrile (MCE-1).

The most prominent feature of the majority of small molecule NRF2 activators is their sulfhydryl reactivity. In 1988, a decade before the discovery of NRF2, Paul Talalay and his colleagues recognized that many structurally diverse NQO1 inducers are Michael reaction acceptors characterized by olefinic (or acetylenic) bonds that are rendered electrophilic by conjugation with electron-withdrawing groups^12^. This was a breakthrough discovery as it identified a common feature of these seemingly unrelated compounds, and importantly, also made possible the prediction of chemical structures with potential inducer activity. To allow testing for new inducers, a highly quantitative and robust bioassay was developed which measures changes in the NQO1 enzyme activity in murine Hepa1c1c7 cells; the Concentration that Doubles the specific activity of NQO1 (CD value) provides a measure of inducer potency^13^. Using this assay, extremely high NQO1 inducer potency, with CD values in the low nanomolar concentration range, was reported for the semisynthetic triterpenoids, such as CDDO (2-cyano-3,12-dioxooleana-1,9(11)-dien-28-oic acid, Fig. 1) and its analogs with cyanoenone functionalities as Michael acceptors^14^. These compounds were derived from a natural product, oleanolic acid (3-β-hydroxyolean-12-en-28-oic acid) and initially identified as highly potent inhibitors of inducible nitric oxide synthase (iNOS)^15–19^. The introduction of the strongly electrophilic cyanoenone functionality into the oleanolic acid skeleton is considered to confer potent NRF2-inducing and anti-inflammatory activities. Currently, the methyl ester analog of CDDO (CDDO-Me, also known as bardoxolone methyl) is being evaluated in Phase II clinical trials for the treatment of pulmonary arterial hypertension (PAH) in the USA (NCT02036970, ClinicalTrials.gov) and diabetic nephropathy in Japan (NCT02316821, ClinicalTrials.gov).

Subsequently, many tricyclic and monocyclic compounds containing cyanoenone functionalities were designed and synthesized, and their potencies were evaluated using the quantitative NQO1 bioassay in Hepa1c1c7 cells. Among these derivatives, the acetylenic tricyclic bis(cyanoenone), (±)-(4b*S*,8a*R*,10a*S*)-10a-ethynyl-4b,8,8-trimethyl-3,7-dioxo-3,4b,7,8,8a,9,10,10a-octahydrophenanthrene-2,6-dicarbonitrile (TBE-31, Fig. 1) is the most potent inducer, active at low and even sub-nanomolar concentrations (CD = 0.9 nM)^20–22^. Both of the cyanoenone functionalities in rings A and C contribute to the inducer activity of TBE-31, and their simultaneous presence within a rigid three-ring system results in exceptionally high inducer potency^23^.

The cyanoenone inducers potently inhibit pro-inflammatory pathways. Thus, exposure to 1 nM of TBE-31 suppresses nitric oxide (NO) production induced by interferon gamma (IFN-γ) in RAW 264.7 cells^20,22^. The oral administration of TBE-31 to rats protects them from aflatoxin-induced hepatocarcinogenesis by reducing the formation of DNA-aflatoxin adducts as well as the size and number of preneoplastic lesions^20^. This effect is mediated through the activation of NRF2 and the inhibition of pro-inflammatory pathways. This study suggests the potential use of TBE-31 as a chemotherapeutic and chemopreventive agent. The role of NRF2 as the mediator of the protective effects of TBE-31 against tumor development is further strengthened by the fact that genetic (by KEAP1 knockdown) or pharmacological (by small quantities of TBE-31, administered topically twice a week) activation of NRF2 reduces inflammation and photocarcinogenesis in the skin of SKH-1 hairless mice exposed to ultraviolet (UV) radiation^24^, and the protective effect of the KEAP1 knockdown is abrogated when NRF2 is deficient^25^. At higher concentrations (in the sub-micromolar range), TBE-31 suppresses non-small cell lung cancer (NSCLC) cell migration and epithelial mesenchymal transition (EMT) by inhibiting TGFβ-dependent actin stress fiber formation and by directly binding to actin, inhibiting its polymerization^26^, suggesting the potential utility of TBE-31 in metastatic disease. Most recently, it was shown that oral administration of TBE-31 to wild type, but not NRF2-knockout mice that had been rendered obese and insulin resistant by consumption of high-fat and high-fructose diet, reversed insulin resistance, and attenuated obesity and liver disease^27^.

The tricyclic cyanoenone MCE-23 (9a-ethynyl-3-oxo-9,9a-dihydro-3*H*-fluorene-2-carbonitrile) (Fig. 1) shares ring C with TBE-31, but lacks the cyanoenone functionality in ring A, and is relatively less potent than TBE-31 as an NQO1 inducer (CD = 41 nM)^28^. The monocyclic cyanoenone MCE-1 (3-ethynyl-3-methyl-6-oxocyclohexa-1,4-dienecarbonitrile) (Fig. 1), which represents ring C of TBE-31 and MCE-23, is also an NQO1 inducer (CD = 22 nM)^23,29^ and has a strong anti-inflammatory activity in RAW 264.7 cells and primary murine macrophage cells^29^. Both TBE-31 and MCE-1 have antidepressant effects in an animal model of inflammation-mediated depression^30^.

TBE-31 binds to KEAP1 covalently and reversibly^23^, and is suitable for chronic *in vivo* administration^31^. TBE-31, MCE-23 and MCE-1 are all highly reactive with sulfhydryl groups as they contain activated Michael acceptors within their structures. By use of the crystalline-sponge method, the crystal structure of the Michael adduct of MCE-23 with a small molecule thiol was determined^32^. *In vitro*, TBE-31 binds to KEAP1 cysteines^23^, but the specific sensor cysteine(s) has not been defined. A recent study has reported that KEAP1 C151 is required for NRF2 activation by a series of pentacyclic and tricyclic cyanoenones^33^. Whether C151 is also the sensor for TBE-31, MCE-23, or MCE-1 is currently unknown. In addition to C151, which is located in the BTB domain of KEAP1, C273 and C288 located in the intervening region, are well-recognized sensor cysteines^34–37^. In the current study, using KEAP1-knockout (KKO) mouse embryonic fibroblast (MEF) cells rescued with wild-type (WT) or cysteine mutants of KEAP1 and primary macrophages isolated from KEAP1-wild type (KEAP1^+/+^) or KEAP1-C151S-mutant (KEAP1^C151S/C151S^) mice, and TBE-31, MCE-23, and MCE-1 as model cyanoenones, we established that C151 is the main cysteine sensor for the cyanoenone class of inducers, irrespective of their molecular size or shape, and further uncovered a previously unrecognized flexibility in the sensing mechanism. Moreover, in primary macrophage cells expressing C151S mutant KEAP1, at low inducer concentrations, the tricyclic cyanoenone TBE-31 is inactive not only as an inducer of the classical NRF2 target NQO1, but also as an inhibitor of lipopolysaccharide-stimulated gene expression of the pro-inflammatory cytokines interleukin 6 (IL6) and interleukin IL1β (IL1β). To our knowledge, this is the first demonstration for the requirement of C151 in KEAP1 for the inhibition of pro-inflammatory gene expression by an NRF2 activator.

## Results

### Cysteine 151 in KEAP1 is the primary sensor for the cyanoenone inducers in MEF cells

Early mutagenesis studies showed that mutants of KEAP1, in which C273 and/or C288 were substituted with either serine or alanine, were inactive in repressing NRF2, rendering the transcription factor constitutively active^35,36,38^. To test whether these cysteines serve as inducer sensors in cells, it was necessary to identify amino acid substitutions, which preserve the repressor function of KEAP1. Using a systematic approach whereby C273 and/or C288 were substituted with each of the natural amino acids, it was found that C273W and C288E mutations do not affect the ability of KEAP1 to repress NRF2^39^. With this information in hand, a new experimental system was developed based on the introduction of an expression vector encoding hemagglutinin (HA)-tagged murine KEAP1 ligated to the PiggyBac transposon system^39^. Expression plasmids encoding wild-type (WT) or cysteine(s) mutant HA-KEAP1 were transfected into KKO MEF cells, and various stable HA-KEAP1 expressing lines were established. Using this system, it was found that the semisynthetic triterpenoid CDDO-imidazolide [CDDO-Im, 1-[2-cyano-3,12-dioxooleana-1,9(11)-dien-28-oyl]imidazole)] (Fig. 1)^17^ and the closely related compound, RTA-408, required C151 for their ability to cause NRF2 stabilization^39,40^, implicating C151 as the sensor cysteine for these pentacyclic cyanoenones.

**Figure 2:**
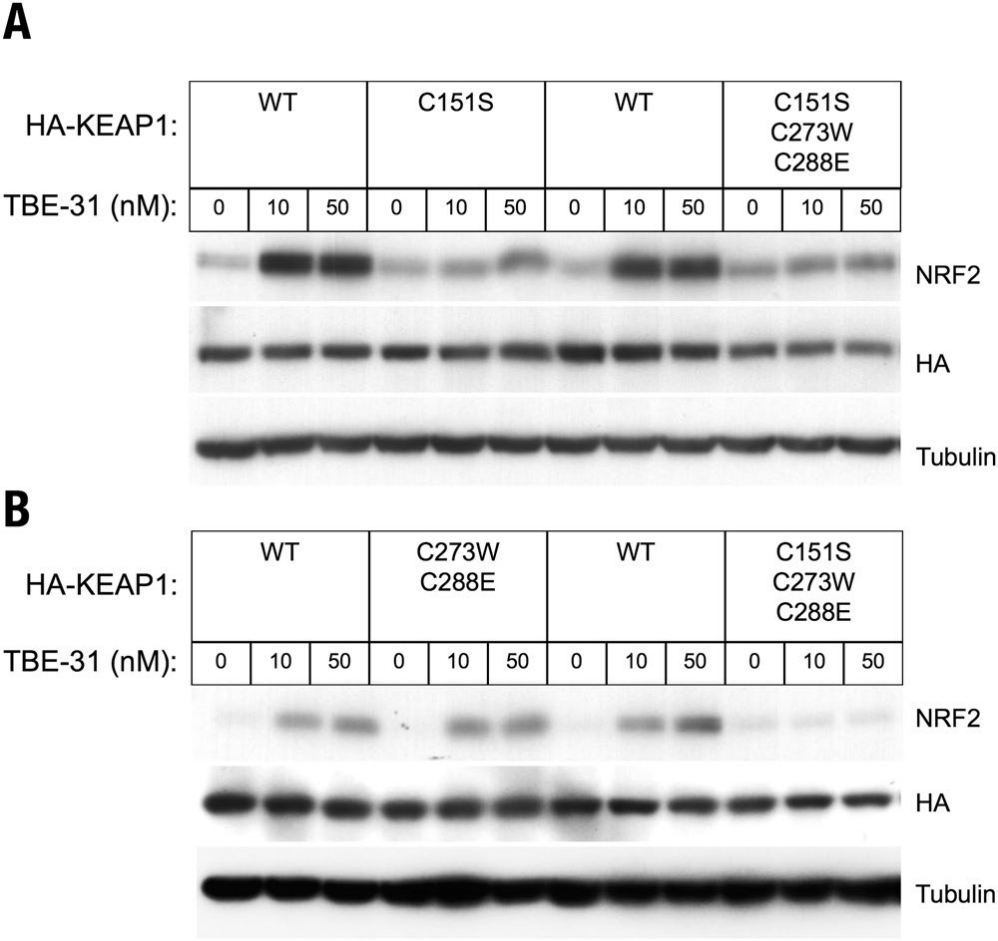
C151 in KEAP1 is the primary sensor for TBE-31 in MEF cells. Western blot analyses of total cell lysates of KEAP1-knockout MEF cells rescued with either wild-type (WT) (**A**,**B**), single cysteine mutant C151S (**A**), double cysteine mutant C273W/C288E (**B**), or triple cysteine mutant C151S/C273W/C288E (**A** and **B**) of mouse N-terminally tagged HA-KEAP1. Cells (3 × 10^5^ per well), growing in 6-well plates, were exposed to vehicle (0.1% DMSO) or TBE-31 for 3 h, after which the cells were lysed. Immunoblotting was performed on cell lysates using antibodies raised against NRF2, HA and α-tubulin.

The tricyclic cyanoenone TBE-31 shares its ring A with the pentacyclic CDDO-Im, but the two compounds differ in size (Fig. 1). To test whether the smaller TBE-31 is sensed by the same or different cysteine(s) in KEAP1, which senses the pentacyclic CDDO-Im and RTA-408, KKO MEF cells stably expressing HA-tagged WT KEAP1 (termed WT-KKO) or its cysteine mutants, namely C151S-KKO, C273W/C288E-KKO, or C151S/C273W/C288E-KKO, were exposed to TBE-31 (10 or 50 nM) for 3 h, and the levels of NRF2 were determined in whole cell lysates by immunoblotting. Because inactivation of KEAP1 leads to NRF2 stabilization, the levels of NRF2 serve as a sensitive readout for KEAP1 activity. An antibody against HA was used to report on the uniformity of the expression levels of WT KEAP1 and its cysteine mutants. In the WT-KKO or the double cysteine mutant C273W/C288E-KKO MEF cells, NRF2 accumulated upon treatment with TBE-31 (Fig. 2A,B). In contrast, under identical experimental conditions, NRF2 did not accumulate in KKO MEF cells harboring the single KEAP1 mutant C151S or the triple KEAP1 mutant C151S/C273W/C288E (Fig. 2A,B). These results show that, similar to the pentacyclic cyanoenones CDDO-Im and RTA-408, C151 is the primary sensor in KEAP1 for the tricyclic TBE-31.

**Figure 3:**
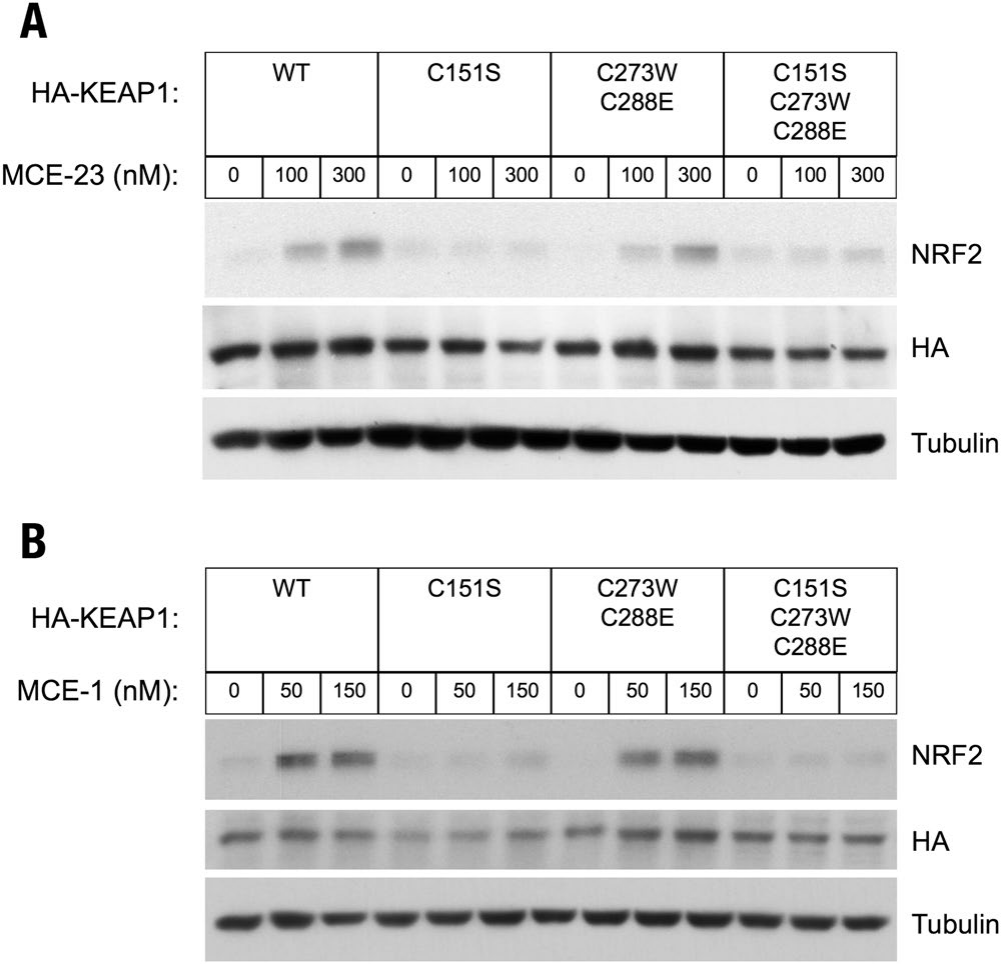
C151 in KEAP1 is the primary sensor for MCE-23 and MCE-1 in MEF cells. Western blot analyses of total cell lysates of KEAP1-knockout MEF cells rescued with either wild-type (WT), single cysteine mutant C151S, double cysteine mutant C273W/C288E or triple cysteine mutant C151S/C273W/C288E of mouse N-terminally tagged HA- KEAP1. Cells (3 × 10^5^ per well), growing in 6-well plates, were exposed to vehicle (0.1% DMSO) (**A**,**B**), MCE-23 (**A**) or MCE-1 (**B**) for 3 h, after which the cells were lysed. Immunoblotting was performed on cell lysates using antibodies raised against NRF2, HA and α-tubulin.

To determine the cysteine sensor(s) of KEAP1 for MCE-23, which in contrast to TBE-31 has only one reactive center, and for MCE-1, which has the smallest size in this cyanoenone set of compounds, KEAP1 KO MEFs rescued with either WT or cysteine mutants of KEAP1, were treated with MCE-23 or MCE-1 for 3 h. In the WT-KKO and the double mutant C273/C288-KKO MEF cells, treatment with either compound caused NRF2 accumulation in a dose-dependent manner (Fig. 3A,B). However, treatment of the single mutant C151S-KKO or the triple mutant C151S/C273W/C288E-KKO MEF cells with MCE-23 or MCE-1 failed to upregulate NRF2, indicating that, similar to the bis(cyanoenone) TBE-31, C151 is the primary sensor in KEAP1 for the cyanoenones MCE-23 and MCE-1. Together with our previous findings, these results show that a pentacyclic compound like CDDO-Im can be reduced in size to a tricyclic compound, such as TBE-31 or MCE-23, which can be further reduced in size to a monocyclic compound like MCE-1, and still retain specificity for the same cysteine sensor in KEAP1, C151. These results further imply that preserving the reactive cyanoenone center is sufficient to confer specificity, and that chemical reactivity (rather than shape or size) is the most important feature of the cyanoenone inducers. These conclusions are supported by data from two recent crystallographic studies. Thus, the crystal structure of the BTB domain of KEAP1 in complex with CDDO was solved^41^, showing that CDDO occupies a shallow groove containing C151, which becomes deeper and more defined upon compound binding. This is in agreement with the recently determined crystal structures of BTB KEAP1 in complex with a tricyclic cyanoenone pyrimidine derivative (TX64014) and of BTB KEAP1 in complex with a tricyclic cyanoenone pyrazole derivative (TX64063)^33^, which share the same cyanoenone function in ring A with the cyanoenones examined in the current study.

### Cysteine 151 in KEAP1 is the primary sensor for the cyanoenone inducers in primary peritoneal macrophages

**Figure 4:**
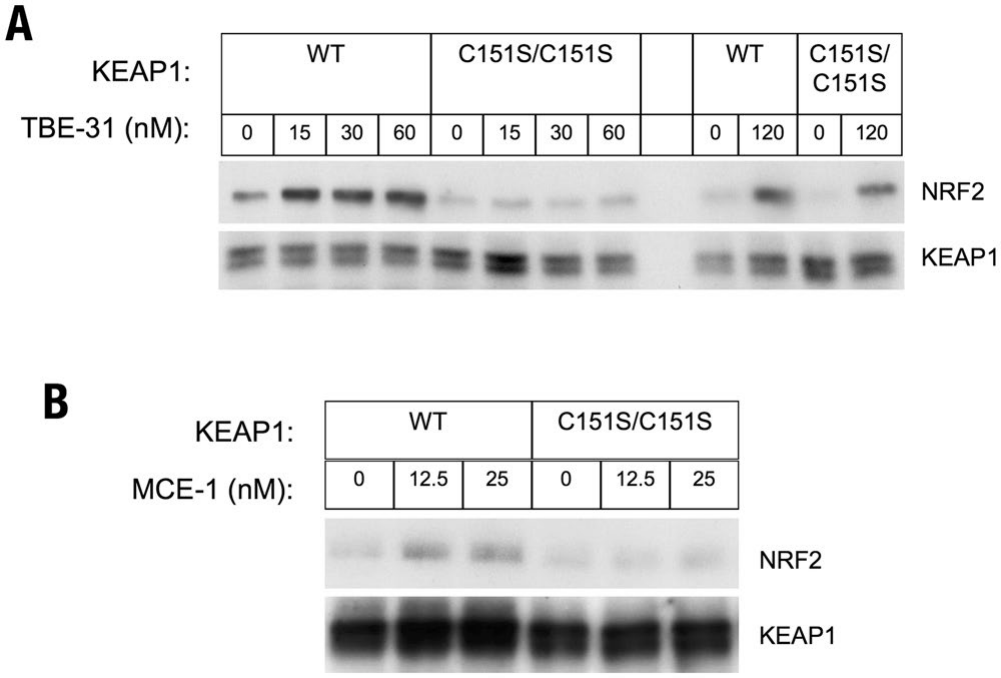
C151 in KEAP1 is the primary sensor for TBE-31 in primary murine peritoneal macrophages. Western blot analysis performed on total cell lysates of primary peritoneal macrophages (10^6^ cells), isolated from WT or KEAP1^C151S/C151S^ mice, that had been treated with vehicle (0.1% DMSO) (**A**,**B**), TBE-31 (**A**) or MCE-1 (**B**) for 3 h. Antibodies raised against NRF2 and KEAP1 were used for detection.

To confirm that our conclusion that C151 is the primary sensor cysteine in KEAP1 for the cyanoenones is not limited to MEF cells, we next isolated primary peritoneal macrophages (PMφ) from WT (KEAP1^+/+^) and KEAP1^C151S/C151S^ mutant mice, and exposed these cells to a range of concentrations (15, 30, 60 or 120 nM) of TBE-31 for 3 h. In the primary WT PMφ cells, NRF2 accumulation was observed at all of the tested concentrations of TBE-31 (Fig. 4A). In contrast, in the primary KEAP1^C151S/C151S^ PMφ cells, NRF2 did not accumulate when the cells were exposed to lower concentrations (15, 30 or 60 nM) of TBE-31 (Fig. 4A). Similar results were obtained after a 3-h exposure to 12.5 or 25 nM of MCE-1 (Fig. 4B). These findings confirm that C151 in KEAP1 is required for NRF2 stabilization by both the tricyclic and the monocyclic cyanoenones. Surprisingly, however, exposure to 120 nM TBE-31 (the highest concentration tested) was able to cause stabilization of NRF2 in both WT and KEAP1^C151S/C151S^ PMφ cells. Together, these results suggest that although C151 is the primary sensor cysteine for TBE-31, when in excess, the inducer is able to also affect other cysteines of KEAP1, and consequently lead to NRF2 activation. Alternatively, it is possible that at high inducer concentrations, TBE-31 causes NRF2 stabilization by inactivating the KEAP1-independent glycogen synthase kinase-3 (GSK3)/β-transducin repeat-containing protein (β-TrCP)-mediated degradation of NRF2^42–44^. However, this is unlikely to occur under our experimental conditions where GSK3 is constitutively phosphorylated (and therefore maintained inactive) due to the growth factors present in the cell culture medium.

### Cysteine 151 in KEAP1 is required for induction of NRF2-mediated transcription by low, but not high concentrations of TBE-31

**Figure 5:**
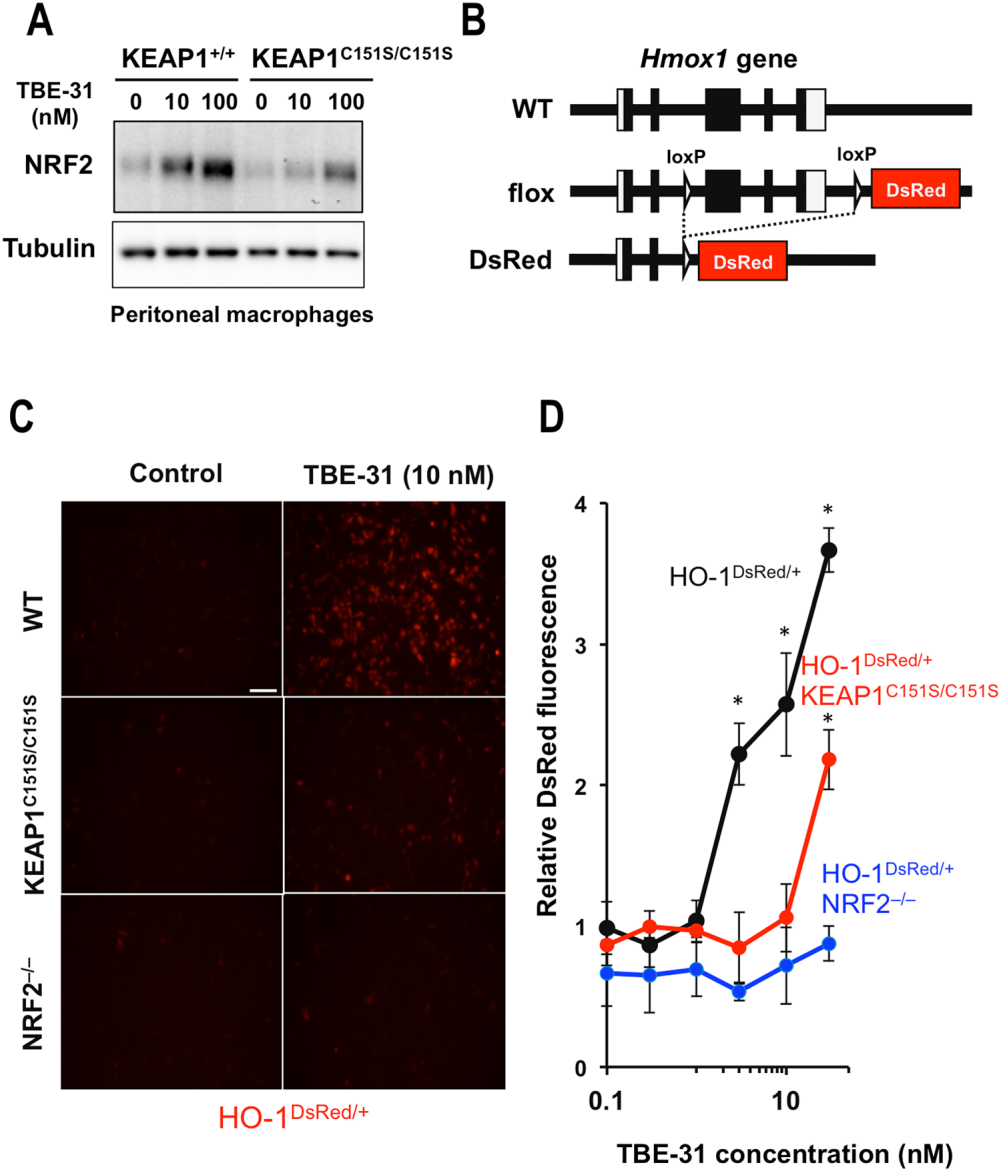
At low inducer concentrations, C151 in KEAP1 is required for the ability of TBE-31 to activate expression of the HO-1 DsRed reporter in primary murine peritoneal macrophages. (**A**) NRF2 levels in total cell lysates of primary peritoneal macrophages isolated from WT or KEAP1^C151S/C151S^ mice, that had been treated with vehicle (0.1% DMSO) or TBE-31 for 3 h. (**B**) Strategy for generating the HO-1^DsRed/+^ reporter mice. Deletion of the floxed region from the mouse *Hmox1* flox allele by the Cre recombinase resulted in generation of the DsRed allele, in which the 3rd to 5th exons encoding an essential domain for the HO-1 activity are deleted, resulting in the expression of a DsRed fusion protein containing the N-terminal 48 amino acids of the HO-1 protein. (**C**) Representative fluorescence microscopy images of primary peritoneal macrophages isolated from WT-, KEAP1^C151S/C151S^- or NRF2^−/−^HO-1^DsRed/+^ reporter mice, that had been treated with vehicle (0.1% DMSO) or 10 nM TBE-31 for 24 h. Scale bar, 100 µm. (**D**) Quantification of the fluorescence of DsRed in primary peritoneal macrophages (n = 4) isolated from mice of the three genotypes following a 24 h-treatment with increasing concentrations of TBE-31. *p < 0.05.

It has been shown previously that TBE-31 is a potent and robust inducer of the NRF2-transcriptional target heme oxygenase 1 (HO-1) in cells and *in vivo*^20^. Having confirmed the requirement for C151 of KEAP1 for stabilization of NRF2 by low (10 nM), but not high (100 nM) concentrations of TBE-31 (Fig. 5A), we next addressed the importance of this cysteine for NRF2-mediated transcription by use of a fluorescent reporter (DsRed) under the control of the promoter of HO-1 (HO-1^DsRed/+^) (Fig. 5B). Treatment with TBE-31 (10 nM) of KEAP1-WT, but not KEAP1^C151S/C151S^ or NRF2-knockout (NRF2^−/−^), cells transfected with this reporter led to an increase in fluorescence intensity (Fig. 5C). A detailed dose-response experiment with quantification of the fluorescence intensity further confirmed the requirement for C151 for induction of NRF2-mediated transcription by TBE-31 concentrations up to 10 nM (Fig. 5D). However, although to a lower magnitude, induction in the absence of C151 was evident at a concentration of TBE-31 of 30 nM (Fig. 5D). These experiments show that, at low inducer concentration, C151 is critical for NRF2 transcriptional activity, but at high inducer concentrations, NRF2 activation can still proceed in the absence of C151.

### Cysteine 151 in KEAP1 is required for the ability of TBE-31 to suppress the LPS-mediated transcriptional activation of interleukin 6 (IL6) and interleukin 1β (IL1β) in primary peritoneal macrophage cells

**Figure 6:**
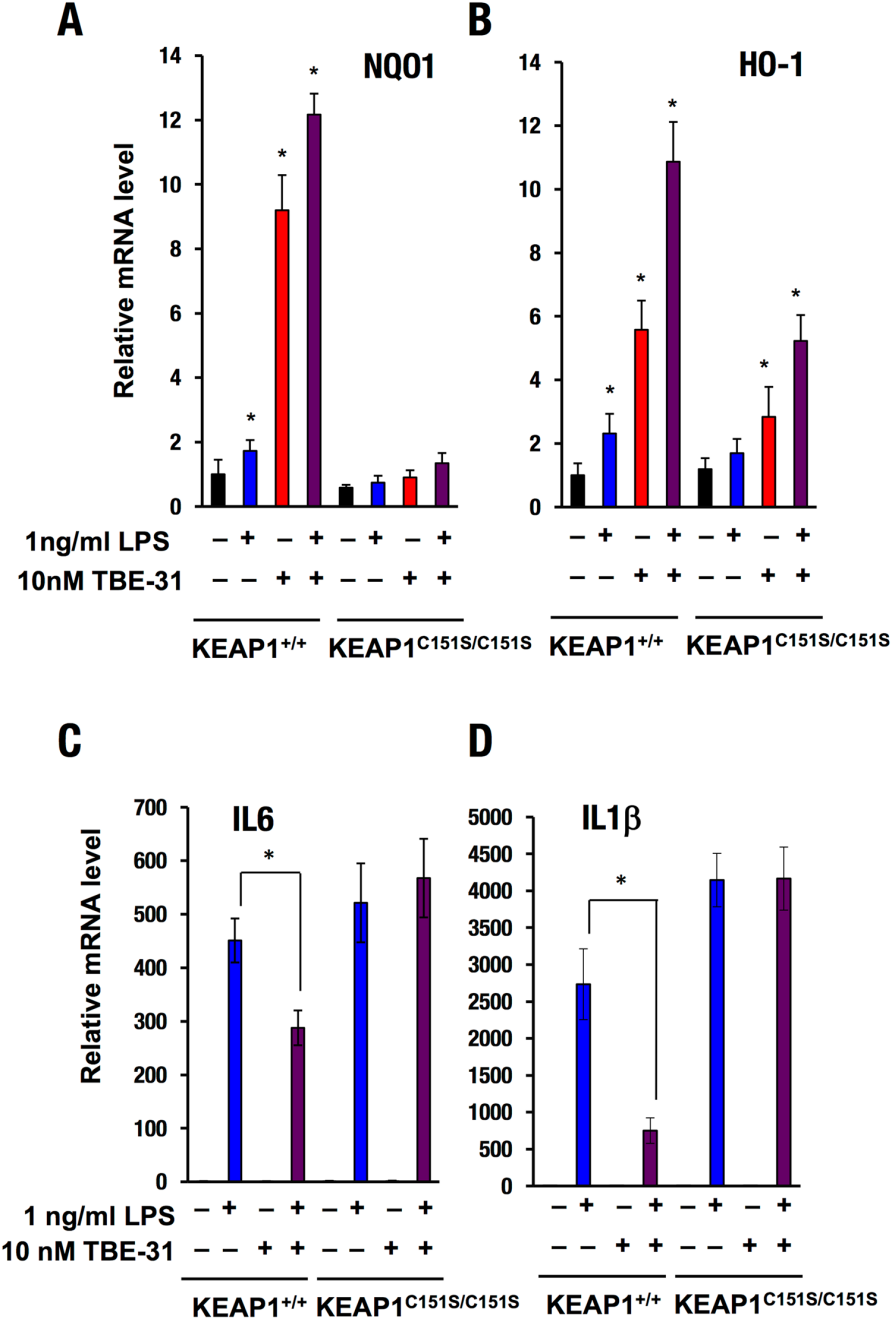
C151 in KEAP1 is required for the ability of TBE-31 to inhibit the gene expression of IL6 and IL1β in primary murine peritoneal macrophages challenged with LPS. mRNA levels for NQO1 (**A**), HO-1 (**B**), IL6 (**C**) and IL1β (**D**) in peritoneal macrophages isolated from WT (KEAP1^+/+^) (n = 7) or KEAP1^C151S/C151S^ (n = 11) mice, that had been treated *ex vivo* with LPS (1 ng/ml) and/or TBE-31 (10 nM) for 4 h. *p < 0.05.

**Figure 7:**
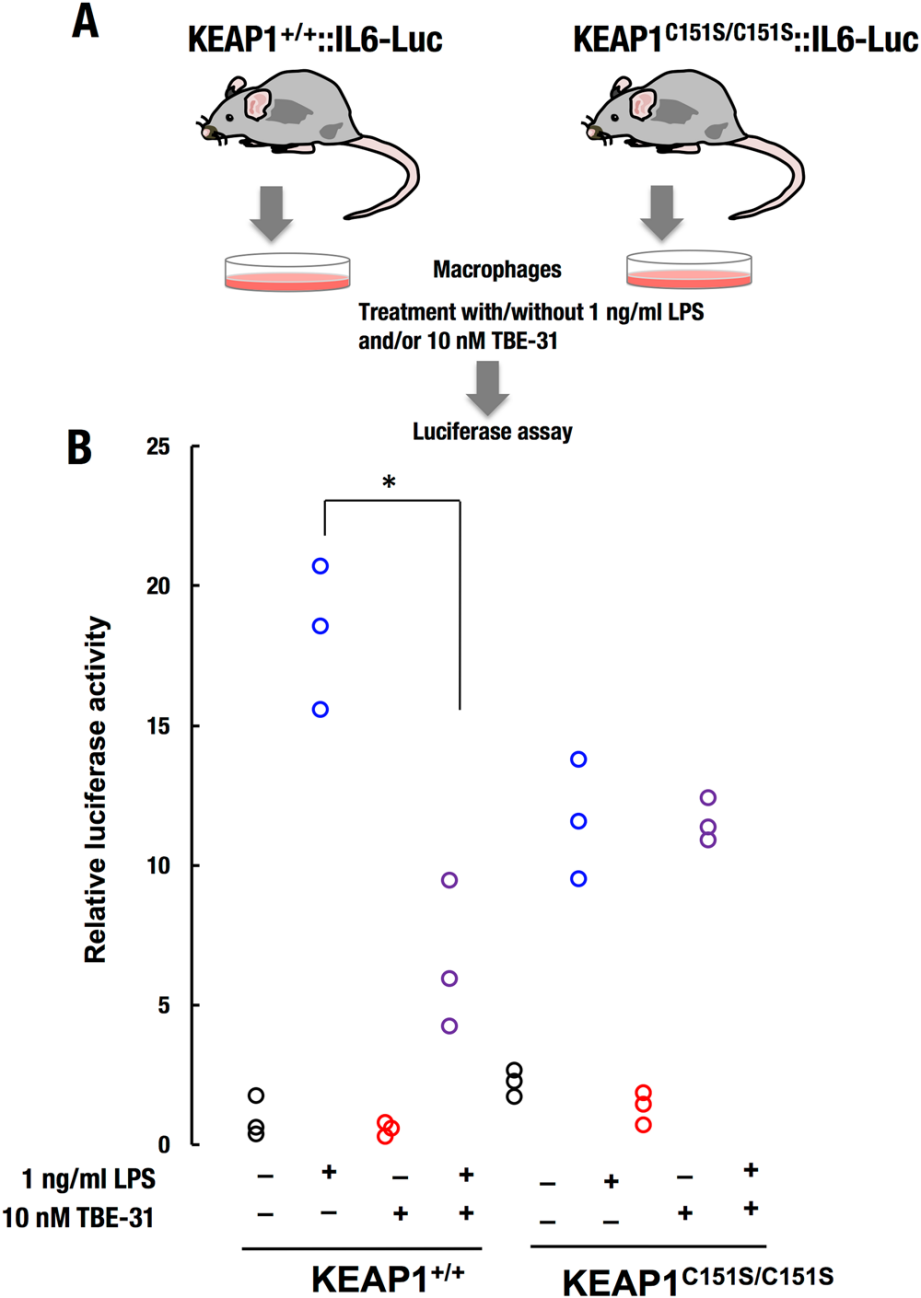
C151 in KEAP1 is required for the ability of TBE-31 to inhibit human *IL6*-luciferase reporter gene expression in primary murine peritoneal macrophages challenged with LPS. (**A**) Primary peritoneal macrophages were isolated from KEAP1^+/+^::hIL6-Luc or KEAP1^C151S/C151S^::hIL6-Luc mice and the cells (n = 3) were treated *ex vivo* with LPS (1 ng/ml) and/or TBE-31 (10 nM) for 4 h. (**A**) The luciferase reporter activity was determined in total cell lysates. *p < 0.05.

Early studies revealed that there is a linear correlation spanning more than 6 orders of magnitude of concentrations between the ability of cyanoenone NRF2 activators to induce NQO1 and to inhibit transcription of pro-inflammatory genes, such as inducible nitric oxide synthase (iNOS)^14^. It was later found that this correlation is valid for NRF2 activators of various chemical classes^45^, implicating NRF2 as the mediator not only for the activation of cytoprotective genes, but also for the inhibition of pro-inflammatory responses. Multiple mechanisms underlie the ability of NRF2 to inhibit the activation of pro-inflammatory genes. An inverse correlation between NRF2 and NF-κB signaling has been observed in numerous experimental systems^27,46–49^. NRF2 activation has been shown to limit the type I interferon response^50,51^. Chromatin immunoprecipitation (ChIP)-seq and ChIP-qPCR analyses have revealed that, in macrophage cells stimulated with lipopolysaccharide (LPS), NRF2 binds to the proximity of the genes encoding IL6 and IL1β, and inhibits the recruitment of RNA Pol II, without affecting the recruitment of the pro-inflammatory transcription factors NF-κB p65 or C/EBPβ^52^. We have previously shown that TBE-31 protects SKH-1 hairless mice against inflammation caused by exposure to solar-simulated UV radiation, in part by inhibiting the transcription of pro-inflammatory genes, such as IL6 and IL1β in the skin of these mice^24^. We therefore next asked whether C151 in KEAP1 was required for the ability of TBE-31 to inhibit the gene expression of IL6 and IL1β. PMφ cells isolated from WT (KEAP1^+/+^) or KEAP1^C151S/C151S^ mutant mice were challenged with LPS (1 ng/ml) with or without TBE-31 (10 nM) for 4 h. In full agreement with the data for the protein levels of NRF2 (Figs 4A and 5A), NRF2 transcriptional activation (as evidenced by the increase in the mRNA levels for NQO1) by TBE-31 only occurred in the KEAP1^+/+^, but not in the KEAP1^C151S/C151S^ PMφ cells (Fig. 6A). Compared to KEAP1^+/+^ cells, the TBE-31-mediated upregulation of the mRNA levels for HO-1, which is transcriptionally regulated by multiple mechanisms^53^, including activation of NRF2, was substantially diminished, although not fully abolished in the KEAP1^C151S/C151S^ PMφ cells (Fig. 6B). Furthermore, the LPS-mediated transcriptional activation of IL6 and IL1β was suppressed by TBE-31 in KEAP1^+/+^ PMφ cells, but not in their KEAP1^C151S/C151S^ counterparts (Fig. 6C,D). The requirement for C151 in KEAP1 for the ability of TBE-31 to suppress the LPS-mediated transcriptional activation of IL6 was further confirmed using PMφ cells isolated from KEAP1^+/+^ and KEAP1^C151S/C151S^ transgenic mice expressing the human IL6-luciferase (*hIL6*-BAC-*Luc*) reporter (Fig. 7A,B). Together, these results show that in macrophage cells, at low inducer concentrations, the anti-inflammatory activity of TBE-31 is due to its reactivity with C151 of KEAP1.

## Discussion

In this study, we show that C151 in KEAP1 is the main cysteine sensor for the cyanoenone class of inducers, irrespective of their molecular size or shape. Moreover, we found that C151 in KEAP1 is required not only for the activity of the tricyclic cyanoenone TBE-31 as an inducer of cytoprotective gene expression, but also as a suppressor of pro-inflammatory responses.

The prevailing view is that KEAP1 is equipped with discreet sensor cysteines, each of which responds to defined types of inducers to trigger NRF2-dependent cytoprotective responses. Here, we found that the concentration of electrophilic cyanoenone NRF2 activators is a critical determinant of their selectivity for the cysteine sensors in KEAP1. Thus, we have identified that, in addition to specificity, there is also flexibility in the sensing mechanism. Our findings have a number of implications. Firstly, they demonstrate the specificity of the sensing mechanism by establishing that C151 is the sensor cysteine in KEAP1 for all of the cyanoenones, irrespective of their shape or size. It is remarkable that a 5-ring compound, such as CDDO-Im, can be reduced in size to a 3-ring compound, such as TBE-31, which can be further reduced in size to a 1-ring compound, such as MCE-1, and still maintain specificity for the same cysteine sensor and consequently, inducer activity. Thus, the cyanoenone functionality is necessary and sufficient for inducer activity. However, other properties of the small molecule play roles in determining inducer potency, such as electron affinity^54^ and enantiospecificity^21,33^, and should be considered in drug design.

Secondly, our findings highlight the intrinsic flexibility and the critical importance of KEAP1 as the principal sensor protein for electrophiles and oxidants: although different specific cysteine sensors have evolved to respond to inducers of different types^35,37,39,55^, there is redundancy among the sensors, which allows the “electrophilic counterattack response”^56^ to proceed even in the event of loss-of-function mutation(s) of specific KEAP1 sensor(s). Such flexibility is extremely important, particularly when the levels of the electrophilic stressor(s) are high, and will prevent glutathione depletion and the ensuing oxidative stress, ensuring cell survival.

Thirdly, our findings explain some experimental results, which at first glance, may be viewed as discrepancies. Thus, it has been reported that stabilization of NRF2 by CDDO-Im (100 nM) is C151-independent^55^, whereas as stated above, in a recent study^39^ we have observed the absolute requirement for C151 in KEAP1 for the CDDO-Im (10 nM)-mediated NRF2 stabilization. Although both studies included an overlapping (30 nM) concentration of the compound, the 10-fold difference in the second concentration of CDDO-Im used in these experiments provides a plausible explanation for these seemingly different conclusions. This redundancy in the sensing mechanism can also explain the finding that *in vitro*, when present at a large (100-fold) stoichiometric excess, CDDO is still able to inhibit the binding of CUL3 to the BTB domain (amino acids 35–235) of KEAP1 bearing a C151S mutation, whereas only 3.3-fold excess is required to achieve the same degree of inhibition (~60%) for the binding of CUL3 to the WT BTB-KEAP1 protein^41^. An excess of inducer in comparison to the main sensor cysteine can also explain the observed partial stabilization of NRF2 and induction of NQO1 in cultured MEF cells from KEAP1^−/−^::Tg^KEAP1C151S^ mutant mice by *tert*-butylhydroquinone (tBHQ)^57^, for which C151 is the main sensor cysteine in KEAP1^35,37,39,55^.

Fourthly, and perhaps most importantly with regard to potential clinical applications, our findings emphasize the critical importance of establishing the precise dose of even the most selective NRF2 activators, such as the cyclic cyanoenones, for maintaining on-target selectivity. Dose precision is of outmost importance for these electrophilic NRF2 activators as such compounds have multiple cellular targets, which become affected in a dose-dependent manner. Thus, CDDO-Im, tBHQ, and guggulsterone have been shown to cause NRF2 stabilization by inhibiting the phosphatase and tensin homolog deleted on chromosome 10 (PTEN), leading to the activation of the phosphoinositide 3-kinase (PI3K)-protein kinase B (PKB)/AKT signaling, phosphorylation of GSK3, and consequently inactivation of the KEAP1-independent GSK3/β- TrCP-mediated degradation of the transcription factor^58–60^. However, this activity does not occur in the presence of growth factor signaling (when GSK3 is phosphorylated and inactive), and requires much higher concentrations of the inducers than those used in our study. Nonetheless, the electrophilic nature of these compounds provides an opportunity for interactions with the cysteine proteome that could affect multiple signaling pathways, such as those regulated by peroxisome proliferator-activated receptor γ (PARPγ), inhibitor of NF-κB kinase (IKK), janus tyrosine kinase/signal transducer and activator of transcription (JAK/STAT), the mechanistic target of rapamycin (mTOR), the retinoic acid receptor (RAR), the estrogen receptor (ER), and the insulin receptor (IR)^61^. These effects might be particularly pronounced under oxidative stress conditions when the levels of reduced glutathione are low.

## Materials and Methods

### Materials

All reagents were of the highest purity available, purchased from common commercial suppliers. The cyanoenone compounds (±)-TBE-31, MCE-23, and MCE-1 were synthesized as described previously^22,28,29^.

### Generation of stable KEAP1-knockout (KKO) KEAP1-rescued MEF cell lines

Immortalized KEAP1-knockout (KKO) MEF cells were grown in low glucose Dulbecco modified Eagle medium (Wako Chemical, Japan) supplemented with 9% fetal bovine serum (FBS) at 37 °C and 5% CO_2_. The PiggyBac transposon system (PB514B-2; System Biosciences, California, USA) was utilized to generate stable cell lines expressing HA (amino acid sequence: YPYDVPDYA)-tagged mouse KEAP1 (HA-KEAP1) or cysteine mutants of HA-KEAP1 by incorporating the cDNA encoding its protein sequence. KKO MEF cells were co-transfected with the PiggyBac expression vector and the plasmid encoding the transposase by electroporation with a double 1,100-V pulse for 30 ms. Forty-eight to 72 h post-transfection, cells were selected with puromycin (2 mg/ml) for one week to 10 days. The expression of red fluorescent protein (RFP) was used as a marker for HA-KEAP1 cDNA incorporation, and single colonies were isolated. These were expanded and tested for the relative expression of endogenous NRF2 and HA-KEAP1 by immunoblotting. For experiments, MEF cells (3 × 10^5^) were plated in each well of a 6 well plate, and 20 to 24 h later, the cells were exposed to either vehicle (0.1% DMSO) or the NRF2 activators for a further 3 h. Following treatment, the cells were subjected to immunoblotting analysis.

### Generation of transgenic mice

All experiments involving animals were approved by the Tohoku University Animal Care Committee and were compliant with the regulations of The Standards for Human Care and Use of Laboratory Animals of Tohoku University (Sendai, Japan) and the Guidelines for Proper Conduct of Animal Experiments of the Ministry of Education, Culture, Sports, Science and Technology of Japan. hIL6-Luc mice were generated using a bacterial artificial chromosome (BAC) clone containing extended flanking sequences of the human interleukin 6 gene (*IL6*) locus, in which the luciferase (Luc) reporter gene is integrated as described^62^. The generation of mice expressing the fluorescent reporter DsRed under the control of the promoter of the mouse *Hmox1* gene (termed HO-1^DsRed/+^mice) will be described in detail elsewhere (manuscript in preparation). Stable KEAP1^C151S/C151S^ knock-in mice were generated using the CRISPR/Cas9 technology. B6D2F1 (C57BL6 × DBA2) super-ovulating female mice were mated with B6D2F1 males, and fertilized embryos were collected. A cocktail of guide RNA (nucleotide sequence: TGGGCGAGAAATGTGTCCTG), Cas9 mRNA, and the donor oligonucleotide were injected into the cytoplasm of fertilized embryos, which were maintained in M16 mouse embryo medium placed in a 37 °C humidified incubator in 5% CO_2_ in air. Subsequently, 15 to 25 embryos were implanted into pseudo-pregnant ICR female mice. The mice containing the mutation on the *Keap1* allele were crossed with WT C57BL6 mice to produce heterozygote animals. Mice that were confirmed heterozygotes were then crossed to produce KEAP1^C151S/C151S^ mice. The KEAP1^C151S/C151S^ mice, which were used in the experiments were maintained for two generations by crossing with WT C57BL6 mice. Genotyping was performed by quantitative real-time PCR (Applied Biosystems). The WT control mice were of C57BL6 genetic background. All information regarding the sequences of the guide RNA, targeting oligonucleotide, and genotyping primers, is available upon request. KEAP1^C151S/C151S^::HO-1^DsRed/+^ and KEAP1^C151S/C151S^::hIL6-Luc mice were generated by crossing KEAP1^C151S/C151S^ mice with HO-1^DsRed/+^ mice and hIL6-Luc mice, respectively. NRF2^−/−^::HO-1^DsRed/+^ mice were generated by crossing NRF2^−/−^mice with HO-1^DsRed/+^ mice.

### Isolation of peritoneal macrophage (PMφ) cells

Mice received 2.0 ml of 4% thioglycolate solution, *i*.*p*. Four days later, the resulting fluid was extracted from the peritoneal cavity, immediately placed on ice and suspended in ice-cold RPMI 1640 medium without serum. The peritoneal macrophage (PMφ) cells were washed and collected by centrifugation (400 × *g* for 10 min at 4 °C). The PMφ cells were then resuspended in RPMI 1640 medium supplemented with 10% FBS, counted, and plated in 6-well plates at a density of 10^6^ cells per well, and placed in a 37 °C humidified incubator in 5% CO_2_ in air. Four hours later, when the PMφ cells had adhered to the plates, they were washed twice with PBS before proceeding with treatments with the inducers as indicated in the figure legends. For the luciferase activity reporter assay, cells were lysed in passive lysis buffer (Promega), the luciferase substrate was added according to the manufacturer’s (Promega) instructions, and the enzyme activity was determined in cell lysates using Lumat LB 9507 tube luminometer (Berthold Technologies).

### Real-time quantitative PCR analysis

RNA was isolated from cells using Sepazol-RNA I Super G RNA Extraction Kit (Nakalai), and cDNA was synthesized from the RNA using ReverTra Ace qPCR RT master mix with gRNA Remover (Toyobo). Real-time quantitative PCR was performed with QuantStudio 6 Real-time PCR System (Applied Biosystems) using THUNDERBIRD SYBR qPCR Mix (Toyobo) or THUNDERBIRD probe qPCR Mix (Toyobo). The primer sequences are listed in Table 1. The mRNA levels for each gene were normalized using hypoxanthine phosphoribosyltransferase (*Hprt*) as an internal control.

**Table 1:** Primer and probe sequences used in this study.

Assay	Gene	Sequence
RT-PCR (TaqMan)	*Nqo1*	Sense AGCTGGAAGCTGCAGACCTG Antisense CCTTTCAGAATGGCTGGCA Probe ATTTCAGTTCCCATTGCAGTGGTTTGGG
	*Hmox1*	Sense GTGATGGAGCGTCCACAGC Antisense TTGGTGGCCTCCTTCAAGG Probe CGACAGCATGCCCCAGGATTTGTC
	*Hprt*	Sense CTGGTGAAAAGGACCTCTCG Antisense TGAAGTACTCATTATAGTCAAGGG Probe ATCCAACAAAGTCTGGCCTGTATCCAAC
RT-PCR (SYBR)	*Il6*	Sense GCTACCAAACTGGATATAATCAGGA Antisense CCAGGTAGCTATGGTACTCCAGAA
	*Il1b*	Sense TGTAATGAAAGACGGCACACC Antisense TCTTCTTTGGGTATTGCTTGG

### Immunoblotting

For immunoblotting analysis of the levels of NRF2 and KEAP1, cells were washed with PBS and lysed by adding to each well of a 6-well plate 200 µl of lysis buffer [100 mM Tris-HCl, pH 6.8; 4% (w/v) sodium dodecyl sulfate (SDS); 20% (v/v) glycerol; 0.001% (w/v) Bromophenol Blue] at room temperature. Following collection of the samples, the lysates were snap-frozen in liquid nitrogen and stored at −80 °C until they were ready to be processed for gel electrophoresis. The lysates were then thawed and subjected to sonication for 30 seconds and subsequently heated at 95 °C for 5 min. After cooling, dithiothreitol (DTT) was added to a final concentration of 20 mM, and the lysates were incubated with the reducing agent at 37 °C for 10 min. The protein concentration of each whole cell lysate was determined by using the BCA protein assay (Thermo Fisher Scientific, Waltham, Massachusetts, USA), and 15–20 μg of total protein were used for the detection of NRF2, HA-KEAP1, KEAP1, and α-tubulin. Proteins were separated by electrophoresis on an 8% SDS–polyacrylamide gel using the BioRad gel electrophoresis system with SDS-running buffer [25 mM Tris-HCl, pH 7.4; 192 mM Glycine; and 0.1% (w/v) SDS], and then electrophoretically transferred to a polyvinylidene difluoride (PVDF) membrane using the Trans-Blot^®^ Turbo™ transfer system (BioRad). After blocking with 10% non-fat milk at room temperature for 1 h or overnight at 4 °C, immunoblotting was performed using the following antibodies for either 1-2 h at room temperature or overnight at 4 °C: rat monoclonal NRF2 antibody^39^ at a dilution of 1:100, rat monoclonal HA antibody (Roche, 3F10, California, USA) at a dilution of 1:1000, or rat monoclonal KEAP1 antibody^39^ at a dilution of 1:100. A mouse monoclonal antibody against α-tubulin (Sigma–Aldrich, DM1A, 1:5000-1:10000 dilution) was used as a loading control. In each case, the data are representative of two to three independent experiments.

### Data analysis

All quantitative data are represented graphically and in the text as mean values ± 1 standard deviation (SD). Student’s t-test was used to test for statistical significance.

### Electronic supplementary material

Supplemental Information

## References

[1] Itoh K (1997). An Nrf2/small Maf heterodimer mediates the induction of phase II detoxifying enzyme genes through antioxidant response elements. Biochem Biophys Res Commun.

[2] Itoh K (1999). Keap1 represses nuclear activation of antioxidant responsive elements by Nrf2 through binding to the amino-terminal Neh2 domain. Genes Dev.

[3] Cullinan SB, Gordan JD, Jin J, Harper JW, Diehl JA (2004). The Keap1-BTB protein is an adaptor that bridges Nrf2 to a Cul3-based E3 ligase: oxidative stress sensing by a Cul3-Keap1 ligase. Mol Cell Biol.

[4] Zhang DD, Lo SC, Cross JV, Templeton DJ, Hannink M (2004). Keap1 is a redox-regulated substrate adaptor protein for a Cul3-dependent ubiquitin ligase complex. Mol Cell Biol.

[5] Kobayashi A (2004). Oxidative stress sensor Keap1 functions as an adaptor for Cul3-based E3 ligase to regulate proteasomal degradation of Nrf2. Mol Cell Biol.

[6] Hayes JD, Dinkova-Kostova AT (2014). The Nrf2 regulatory network provides an interface between redox and intermediary metabolism. Trends Biochem Sci.

[7] Kensler TW, Wakabayashi N, Biswal S (2007). Cell survival responses to environmental stresses via the Keap1-Nrf2-ARE pathway. Annu Rev Pharmacol Toxicol.

[8] Suzuki T, Motohashi H, Yamamoto M (2013). Toward clinical application of the Keap1-Nrf2 pathway. Trends in pharmacological sciences.

[9] Dinkova-Kostova AT, Kostov RV, Canning P (2017). Keap1, the cysteine-based mammalian intracellular sensor for electrophiles and oxidants. Arch Biochem Biophys.

[10] Sihvola V, Levonen AL (2017). Keap1 as the redox sensor of the antioxidant response. Arch Biochem Biophys.

[11] Dinkova-Kostova AT, Talalay P (2010). NAD(P)H:quinone acceptor oxidoreductase 1 (NQO1), a multifunctional antioxidant enzyme and exceptionally versatile cytoprotector. Arch Biochem Biophys.

[12] Talalay P, De Long MJ, Prochaska HJ (1988). Identification of a common chemical signal regulating the induction of enzymes that protect against chemical carcinogenesis. Proc Natl Acad Sci USA.

[13] Prochaska HJ, Santamaria AB (1988). Direct measurement of NAD(P)H:quinone reductase from cells cultured in microtiter wells: a screening assay for anticarcinogenic enzyme inducers. Anal Biochem.

[14] Dinkova-Kostova AT (2005). Extremely potent triterpenoid inducers of the phase 2 response: correlations of protection against oxidant and inflammatory stress. Proc Natl Acad Sci USA.

[15] Honda T (1998). Design and synthesis of 2-cyano-3,12-dioxoolean-1,9-dien-28-oic acid, a novel and highly active inhibitor of nitric oxide production in mouse macrophages. Bioorg Med Chem Lett.

[16] Suh N (1999). A novel synthetic oleanane triterpenoid, 2-cyano-3,12-dioxoolean-1,9-dien-28-oic acid, with potent differentiating, antiproliferative, and anti-inflammatory activity. Cancer Res.

[17] Honda T (2000). Synthetic oleanane and ursane triterpenoids with modified rings A and C: a series of highly active inhibitors of nitric oxide production in mouse macrophages. J Med Chem.

[18] Honda T (2002). A novel dicyanotriterpenoid, 2-cyano-3,12-dioxooleana-1,9(11)-dien-28-onitrile, active at picomolar concentrations for inhibition of nitric oxide production. Bioorg Med Chem Lett.

[19] Favaloro FG (2002). Design and synthesis of tricyclic compounds with enone functionalities in rings A and C: a novel class of highly active inhibitors of nitric oxide production in mouse macrophages. J Med Chem.

[20] Liby K (2008). A novel acetylenic tricyclic bis-(cyano enone) potently induces phase 2 cytoprotective pathways and blocks liver carcinogenesis induced by aflatoxin. Cancer Res.

[21] Honda T (2007). Novel tricyclic compounds having acetylene groups at C-8a and cyano enones in rings A and C: highly potent anti-inflammatory and cytoprotective agents. J Med Chem.

[22] Honda T (2011). Tricyclic compounds containing nonenolizable cyano enones. A novel class of highly potent anti-inflammatory and cytoprotective agents. J Med Chem.

[23] Dinkova-Kostova AT (2010). An exceptionally potent inducer of cytoprotective enzymes: elucidation of the structural features that determine inducer potency and reactivity with Keap1. J Biol Chem.

[24] Knatko EV (2015). Nrf2 Activation Protects against Solar-Simulated Ultraviolet Radiation in Mice and Humans. Cancer Prev Res (Phila).

[25] Knatko EV, Higgins M, Fahey JW, Dinkova-Kostova AT (2016). Loss of Nrf2 abrogates the protective effect of Keap1 downregulation in a preclinical model of cutaneous squamous cell carcinoma. Scientific reports.

[26] Chan E, Saito A, Honda T, Di Guglielmo GM (2016). The acetylenic tricyclic bis(cyano enone), TBE-31, targets microtubule dynamics and cell polarity in migrating cells. Biochim Biophys Acta.

[27] Sharma RS (2018). Experimental Nonalcoholic Steatohepatitis and Liver Fibrosis Are Ameliorated by Pharmacologic Activation of Nrf2 (NF-E2 p45-Related Factor 2). Cell Mol Gastroenterol Hepatol.

[28] Li W (2015). New Monocyclic, Bicyclic, and Tricyclic Ethynylcyanodienones as Activators of the Keap1/Nrf2/ARE Pathway and Inhibitors of Inducible Nitric Oxide Synthase. J Med Chem.

[29] Zheng S (2012). Synthesis, chemical reactivity as Michael acceptors, and biological potency of monocyclic cyanoenones, novel and highly potent anti-inflammatory and cytoprotective agents. J Med Chem.

[30] Yao W (2016). Antidepressant effects of TBE-31 and MCE-1, the novel Nrf2 activators, in an inflammation model of depression. Eur J Pharmacol.

[31] Kostov RV (2015). Pharmacokinetics and pharmacodynamics of orally administered acetylenic tricyclic bis(cyanoenone), a highly potent Nrf2 activator with a reversible covalent mode of action. Biochem Biophys Res Commun.

[32] Duplan V, Hoshino M, Li W, Honda T, Fujita M (2016). *In Situ* Observation of Thiol Michael Addition to a Reversible Covalent Drug in a Crystalline Sponge. Angew Chem Int Ed Engl.

[33] Huerta C (2016). Characterization of novel small-molecule NRF2 activators: Structural and biochemical validation of stereospecific KEAP1 binding. Biochim Biophys Acta.

[34] Dinkova-Kostova AT (2002). Direct evidence that sulfhydryl groups of Keap1 are the sensors regulating induction of phase 2 enzymes that protect against carcinogens and oxidants. Proc Natl Acad Sci USA.

[35] Zhang DD, Hannink M (2003). Distinct cysteine residues in Keap1 are required for Keap1-dependent ubiquitination of Nrf2 and for stabilization of Nrf2 by chemopreventive agents and oxidative stress. Mol Cell Biol.

[36] Levonen AL (2004). Cellular mechanisms of redox cell signalling: role of cysteine modification in controlling antioxidant defences in response to electrophilic lipid oxidation products. Biochem J.

[37] McMahon M, Lamont DJ, Beattie KA, Hayes JD (2010). Keap1 perceives stress via three sensors for the endogenous signaling molecules nitric oxide, zinc, and alkenals. Proc Natl Acad Sci USA.

[38] Wakabayashi N (2004). Protection against electrophile and oxidant stress by induction of the phase 2 response: fate of cysteines of the Keap1 sensor modified by inducers. Proc Natl Acad Sci USA.

[39] Saito R (2015). Characterizations of Three Major Cysteine Sensors of Keap1 in Stress Response. Mol Cell Biol.

[40] Shekh-Ahmad T (2018). KEAP1 inhibition is neuroprotective and suppresses the development of epilepsy. Brain.

[41] Cleasby A (2014). Structure of the BTB domain of Keap1 and its interaction with the triterpenoid antagonist CDDO. PLoS One.

[42] Rada P (2011). SCF/{beta}-TrCP promotes glycogen synthase kinase 3-dependent degradation of the Nrf2 transcription factor in a Keap1-independent manner. Mol Cell Biol.

[43] Rada P (2012). Structural and functional characterization of Nrf2 degradation by the glycogen synthase kinase 3/beta-TrCP axis. Mol Cell Biol.

[44] Chowdhry S (2013). Nrf2 is controlled by two distinct beta-TrCP recognition motifs in its Neh6 domain, one of which can be modulated by GSK-3 activity. Oncogene.

[45] Liu H, Dinkova-Kostova AT, Talalay P (2008). Coordinate regulation of enzyme markers for inflammation and for protection against oxidants and electrophiles. Proc Natl Acad Sci USA.

[46] Thimmulappa RK (2006). Nrf2 is a critical regulator of the innate immune response and survival during experimental sepsis. J Clin Invest.

[47] Chowdhry S (2010). Loss of Nrf2 markedly exacerbates nonalcoholic steatohepatitis. Free Radic Biol Med.

[48] Cuadrado A, Martin-Moldes Z, Ye J, Lastres-Becker I (2014). Transcription factors NRF2 and NF-kappaB are coordinated effectors of the Rho family, GTP-binding protein RAC1 during inflammation. J Biol Chem.

[49] Li W (2008). Activation of Nrf2-antioxidant signaling attenuates NFkappaB-inflammatory response and elicits apoptosis. Biochem Pharmacol.

[50] Olagnier D (2017). Activation of Nrf2 Signaling Augments Vesicular Stomatitis Virus Oncolysis via Autophagy-Driven Suppression of Antiviral Immunity. Mol Ther.

[51] Mills EL (2018). Itaconate is an anti-inflammatory metabolite that activates Nrf2 via alkylation of KEAP1. Nature.

[52] Kobayashi EH (2016). Nrf2 suppresses macrophage inflammatory response by blocking proinflammatory cytokine transcription. Nature communications.

[53] Alam J, Igarashi K, Immenschuh S, Shibahara S, Tyrrell RM (2004). Regulation of heme oxygenase-1 gene transcription: recent advances and highlights from the International Conference (Uppsala, 2003) on Heme Oxygenase. Antioxid Redox Signal.

[54] Bensasson RV (2016). Electron affinity of tricyclic, bicyclic, and monocyclic compounds containing cyanoenones correlates with their potency as inducers of a cytoprotective enzyme. Bioorg Med Chem Lett.

[55] Takaya K (2012). Validation of the multiple sensor mechanism of the Keap1-Nrf2 system. Free Radic Biol Med.

[56] Prestera T, Zhang Y, Spencer SR, Wilczak CA, Talalay P (1993). The electrophile counterattack response: protection against neoplasia and toxicity. Adv Enzyme Regul.

[57] Yamamoto T (2008). Physiological significance of reactive cysteine residues of Keap1 in determining Nrf2 activity. Mol Cell Biol.

[58] Rojo AI (2014). The PTEN/NRF2 axis promotes human carcinogenesis. Antioxid Redox Signal.

[59] Pitha-Rowe I, Liby K, Royce D, Sporn M (2009). Synthetic triterpenoids attenuate cytotoxic retinal injury: cross-talk between Nrf2 and PI3K/AKT signaling through inhibition of the lipid phosphatase PTEN. Invest Ophthalmol Vis Sci.

[60] Almazari I (2012). Guggulsterone induces heme oxygenase-1 expression through activation of Nrf2 in human mammary epithelial cells: PTEN as a putative target. Carcinogenesis.

[61] Yore MM, Kettenbach AN, Sporn MB, Gerber SA, Liby KT (2011). Proteomic analysis shows synthetic oleanane triterpenoid binds to mTOR. PLoS One.

[62] Hayashi M (2015). Whole-Body *In Vivo* Monitoring of Inflammatory Diseases Exploiting Human Interleukin 6-Luciferase Transgenic Mice. Mol Cell Biol.

