# Author Correction: C151 in KEAP1 is the main cysteine sensor for the cyanoenone class of NRF2 activators, irrespective of molecular size or shape

**DOI:** 10.1038/s41598-024-55265-5

**Published:** 2024-02-27

**Authors:** Sharadha Dayalan Naidu, Aki Muramatsu, Ryota Saito, Soichiro Asami, Tadashi Honda, Tomonori Hosoya, Ken Itoh, Masayuki Yamamoto, Takafumi Suzuki, Albena T. Dinkova-Kostova

**Affiliations:** 1 Jacqui Wood Cancer Centre, Division of Cancer Research, School of Medicine, University of Dundee, Dundee, Scotland, UK; 2 Department of Medical Biochemistry, Tohoku University Graduate School of Medicine, Aoba-Ku, Sendai, Japan; 3 Department of Chemistry and Institute of Chemical Biology & Drug Discovery, Stony Brook University, Stony Brook, NY 11794-3400 USA; 4 Department of Stress Response Science, Hirosaki University Graduate School of Medicine, Hirosaki, Japan; 5 Department of Pharmacology and Molecular Sciences and Department of Medicine, Johns Hopkins University School of Medicine, Baltimore, MD USA

Correction to: *Scientific Reports* 10.1038/s41598-018-26269-9, published online 23 May 2018

This Article contains an error in Figure 3.

As a result of an error during the preparation of the figures for this Article, the western blots shown in Figure 3A and 3B contained an additional lane for the protein Tubulin. This is because an additional sample was loaded in the last lane of the gel to prevent potential stretching of the gel in this lane during electrophoresis if left empty. It was subsequently left uncropped from the tubulin blot shown in the published figure.

The corrected Figure 3 and its accompanying legend appear below.

**Figure 3:**
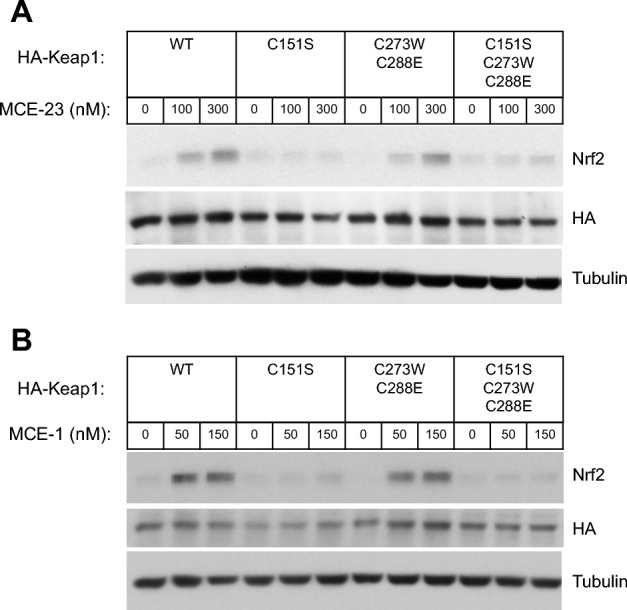
C151 in KEAP1 is the primary sensor for MCE-23 and MCE-1 in MEF cells. Western blot analyses of total cell lysates of KEAP1-knockout MEF cells rescued with either wild-type (WT), single cysteine mutant C151S, double cysteine mutant C273W/C288E or triple cysteine mutant C151S/C273W/C288E of mouse N-terminally tagged HA-KEAP1. Cells (3 × 10^5^ per well), growing in 6-well plates, were exposed to vehicle (0.1% DMSO) (**A**,**B**), MCE-23 (**A**) or MCE-1 (**B**) for 3 h, after which the cells were lysed. Immunoblotting was performed on cell lysates using antibodies raised against NRF2, HA and α-tubulin.

