# Supplementary material for: C151 in KEAP1 is the main cysteine sensor for the cyanoenone class of NRF2 activators, irrespective of molecular size or shape
Source: Sci Rep. 2018 May 23;8:8037. doi: 10.1038/s41598-018-26269-9 (PMC5966396; doi:10.1038/s41598-018-26269-9)

Supplementary Information

C151 in KEAP1 is the main cysteine sensor for the cyanoenone class of NRF2 activators, irrespective of molecular size or shape

Sharadha Dayalan Naidu, Aki Muramatsu, Ryota Saito, Soichiro Asami, Tadashi Honda, Tomonori Hosoya, Ken Itoh, Masayuki Yamamoto, Takafumi Suzuki, and Albena T. Dinkova-Kostova

This fine contains all full-length blots.

FIG 2A

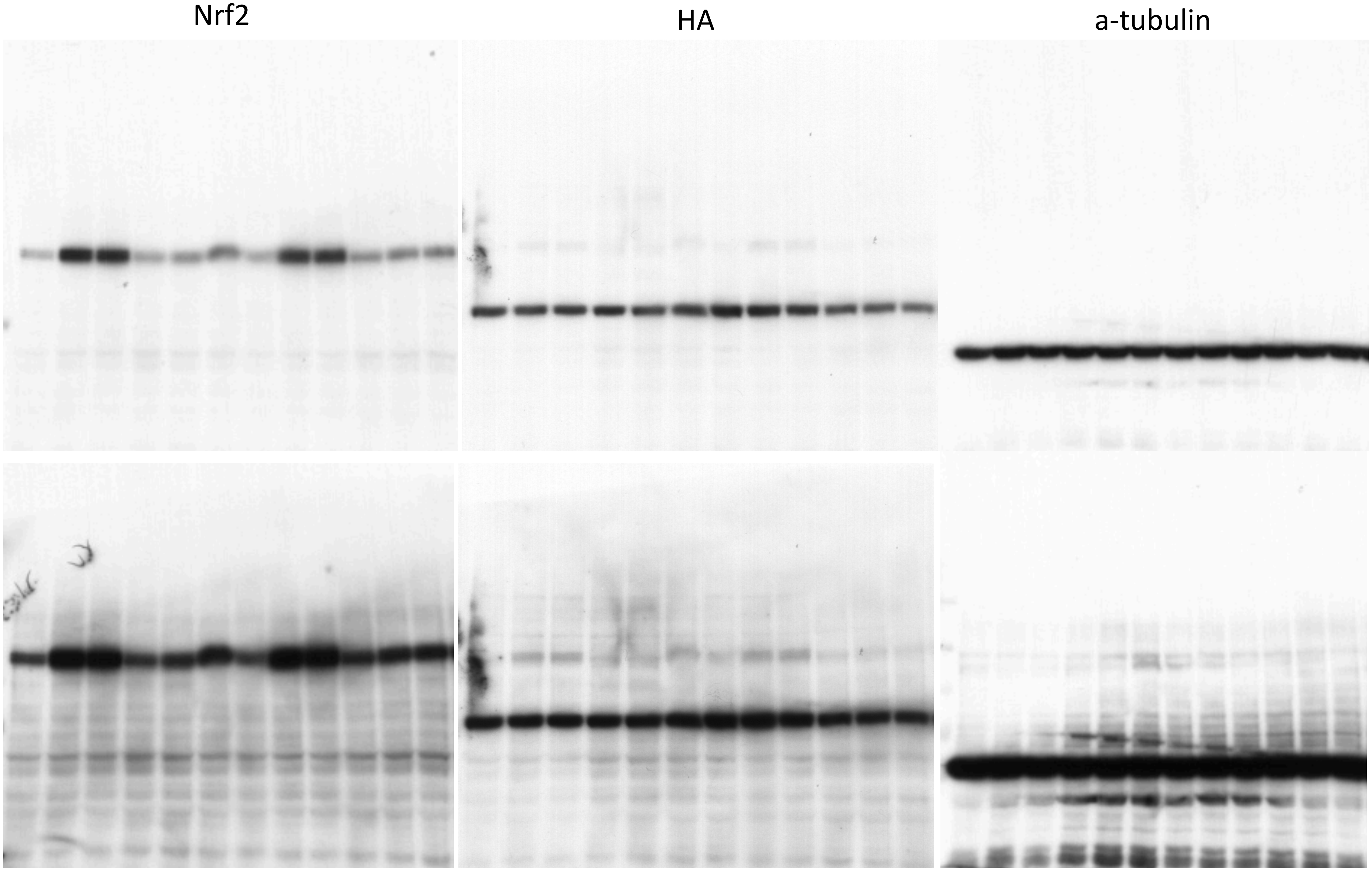

FIG 2B

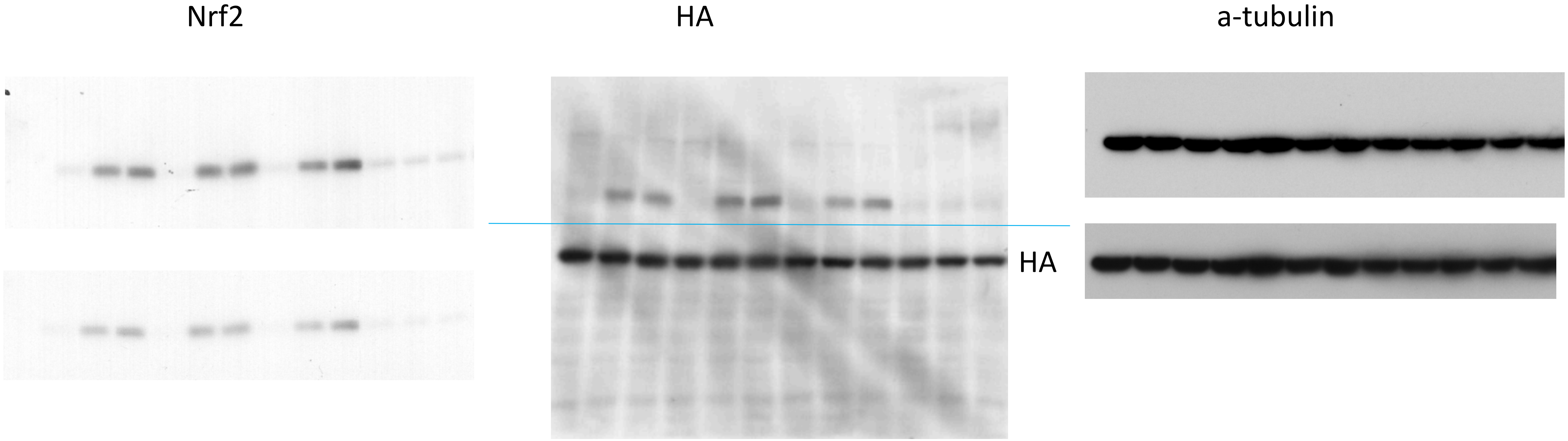

FIG 3A

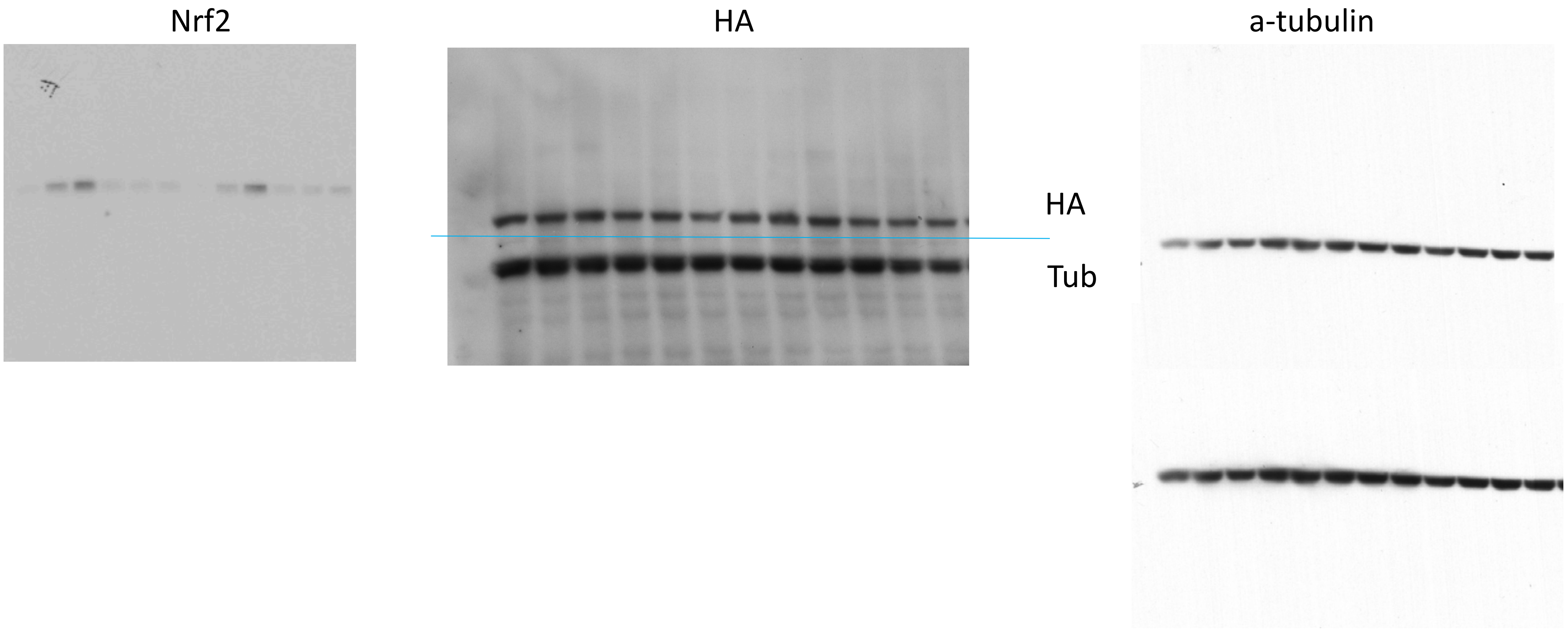

FIG 3B

Nrf2

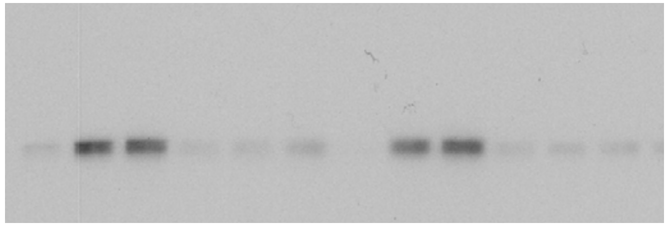

HA

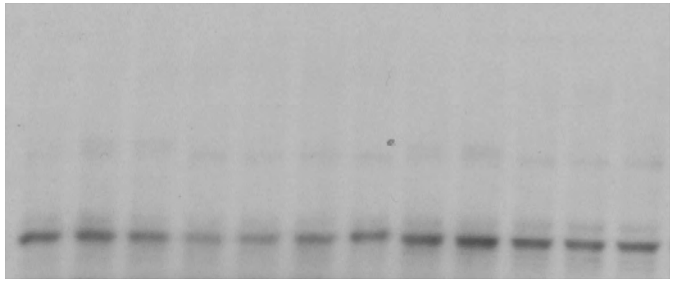

a-tubulin

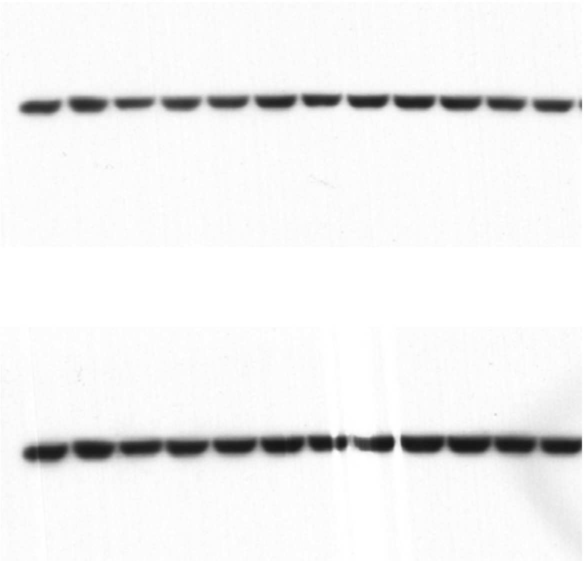

FIG 4A

Nrf2

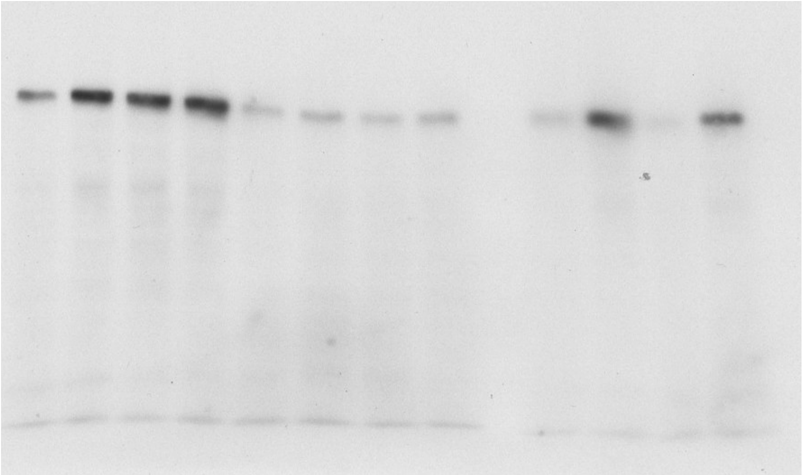

Keap1

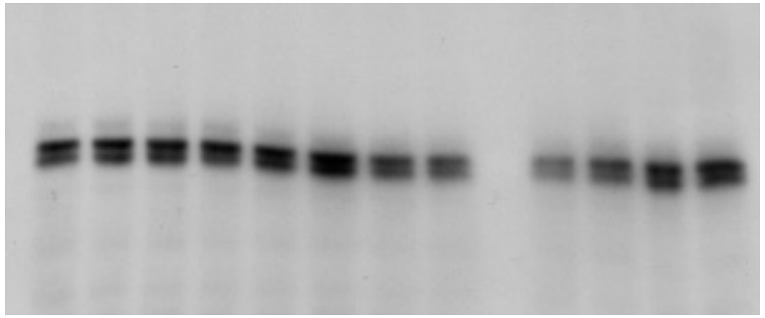

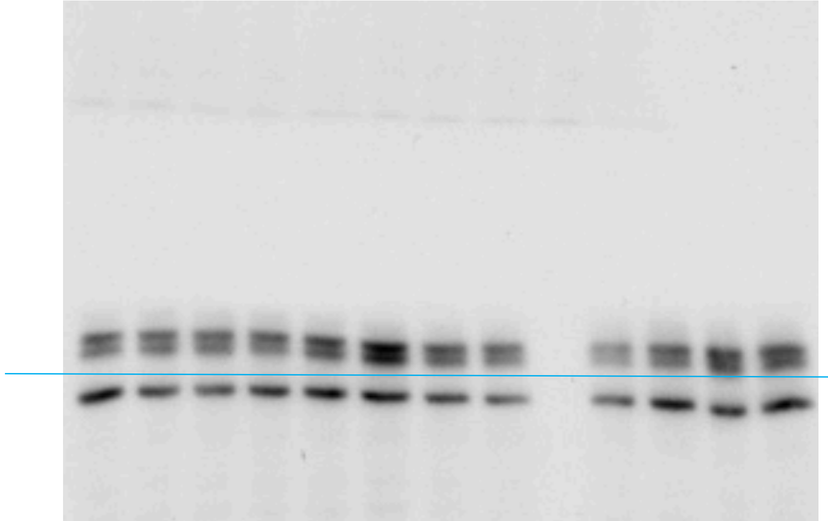

Keap1

FIG 4B

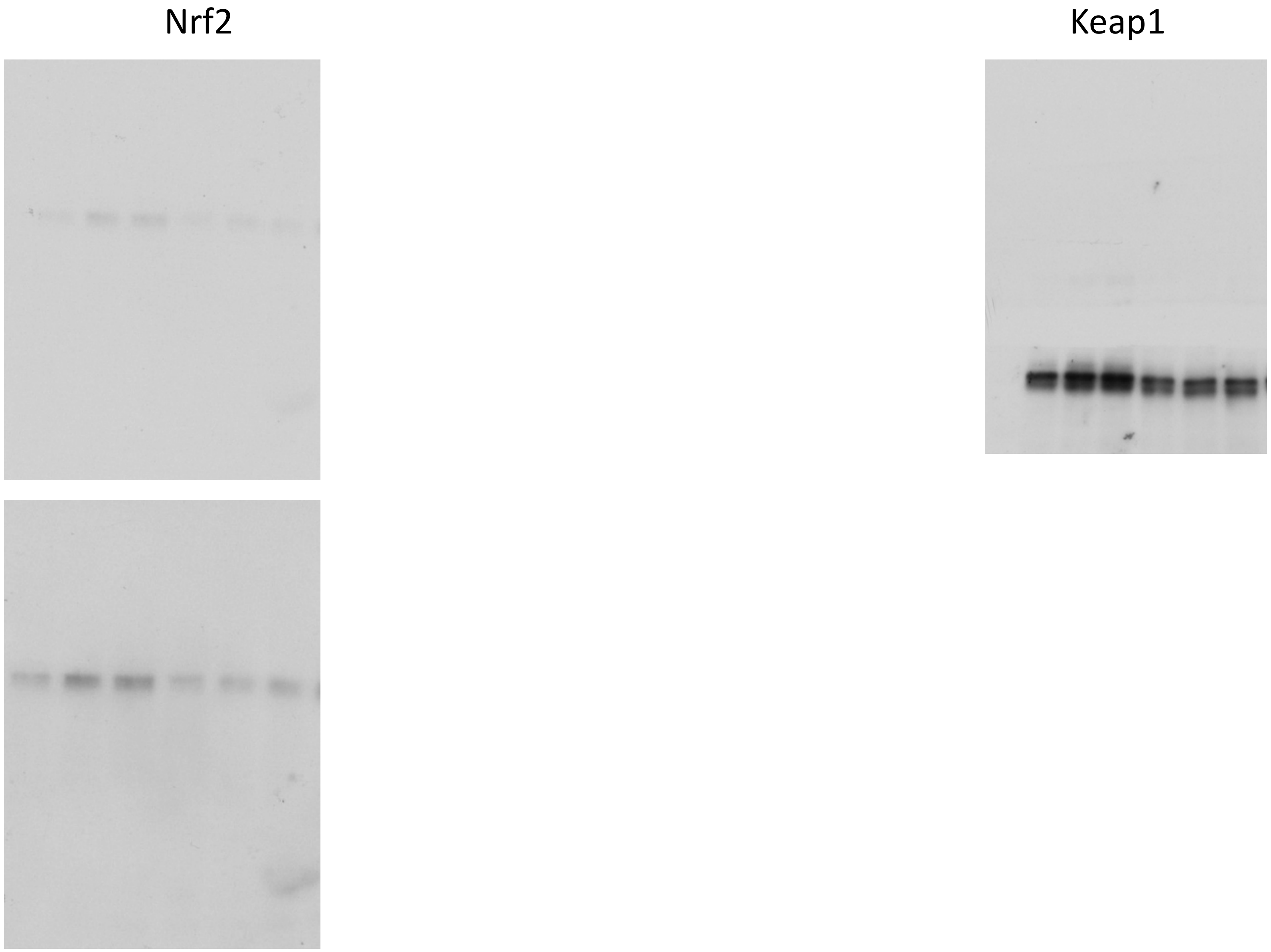

FIG 5A

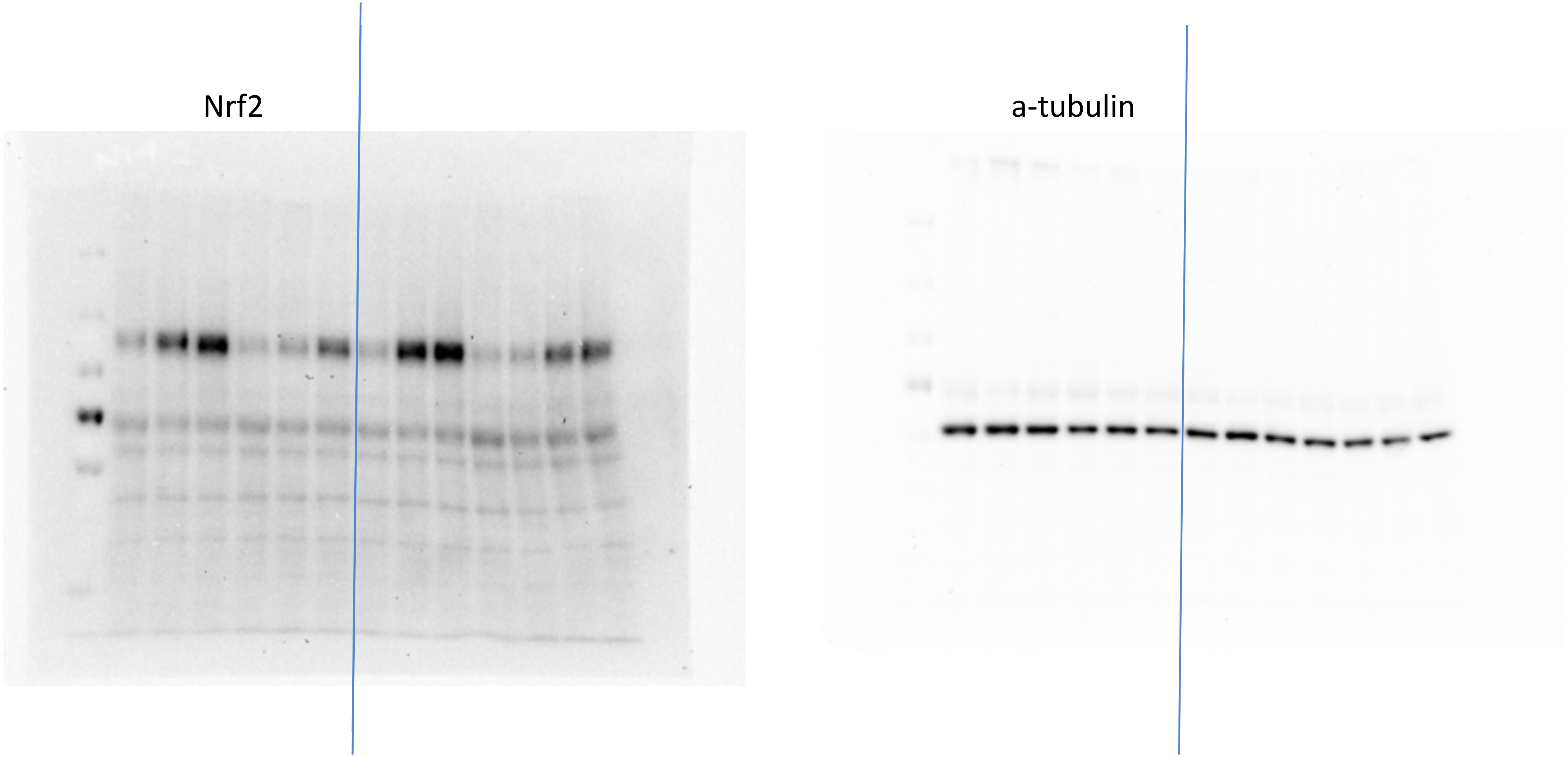

Supplement: Supplementary file 1 — Supplemental Information [file 41598_2018_26269_MOESM1_ESM.pdf]
